# Tongxinluo mitigates atherogenesis by regulating angiogenic factors and inhibiting vasa vasorum neovascularization in apolipoprotein E-deficient mice

**DOI:** 10.18632/oncotarget.7477

**Published:** 2016-02-18

**Authors:** Lianyue Ma, Mei Ni, Panpan Hao, Huixia Lu, Xiaoyan Yang, Xingli Xu, Cheng Zhang, Shanying Huang, Yuxia Zhao, Xiaoling Liu, Yun Zhang

**Affiliations:** ^1^ The Key Laboratory of Cardiovascular Remodeling and Function Research, Chinese Ministry of Education and Chinese Ministry of Health, Qilu Hospital, Shandong University, Shandong 250012, P.R. China; ^2^ The State and Shandong Province Joint Key Laboratory of Translational Cardiovascular Medicine, Qilu Hospital, Shandong University, Shandong 250012, P.R. China

**Keywords:** atherogenesis, vasa vasorum, neovascularization, Tongxinluo

## Abstract

Vasa vasorum (VV) neovascularization contributes to atherogenesis and its expansion and distribution is correlated with intraplaque expression of angiogenic factors. The present study investigated the roles of Tongxinluo (TXL), a traditional Chinese medication, on VV proliferation and atherogenesis. *In vitro*, TXL pre-treatment reversed the tumor necrosis factor-a (TNF-a) induced expression of vascular endothelial growth factor A (VEGF-A) and angiopoietin-1 (ANGPT-1) but not ANGPT-2, leading to increased ratio of ANGPT-1 to ANGPT-2. Consistently, TXL treatment (at a dosage of 0.38, 0.75, 1.5 g/kg/d, respectively) decreased the expression of VEGF-A while increased that of ANGPT-1 in early atherosclerotic lesions of apolipoprotein E deficient (apoE−/−) mice. On aortic ring assay, microvessels sprouting from aortas were significantly inhibited in TXL-treated mice. Moreover, VV neovascularization in plaques was markedly reduced with TXL treatment. Histological and morphological analysis demonstrated that TXL treatment reduced plaque burden, plaque size and changed the plaque composition. These data suggest that TXL inhibits early atherogenesis through regulating angiogenic factor expression and inhibiting VV proliferation in atherosclerotic plaque. Our study shed new light on the anti-atherosclerotic effect of TXL.

## INTRODUCTION

Acute myocardial syndrome (ACS) triggered by atherosclerotic plaque hemorrhage or plaque rupture accounts for substantial morbidity and mortality worldwide [1, 2]. Given the prevalence and poor prognosis of atherosclerosis, strategies to prevent or control its development are of crucial importance.

Angiogenesis is a complex and multistep process of forming new microvessels from pre-existing ones. Vasa vasorum (VV) neovascularization is closely associated with atherogenesis [3–5]. The extent and distribution of VV neovascularization in atherosclerotic plaques is regulated by numerous angiogenic factors. Among these, the vascular endothelial growth factor (VEGF) family and angiopoietin/Tie system play prominent roles. VEGF-A, the major VEGF subtype, is detected at all stages of human coronary atherosclerosis and promotes atherosclerosis progression in apoE/apoB^100^ double-deficient mice and rabbits [6, 7]. Angiopoietin 1 (ANGPT-1), a member of the angiopoietin/Tie system, blocks VEGF-induced vascular permeability and stabilizes the interaction between endothelial cells and supporting cells, whereas, ANGPT-2, an ANGPT-1 antagonist, leads to vascular destabilization or regression [8–10]. Reduced ANGPT-1 and increased ANGPT-2 levels are observed in vulnerable plaques of human carotid artery [11]. In the atherosclerotic plaques with high microvessel density, the ratio of ANGPT-1 to ANGPT-2 is in favor of ANGPT-2 [12].

Tongxinluo (TXL), a traditional Chinese medication, has been widely used to treat angina pectoris for decades [13]. However, the pharmacological mechanism of TXL in coronary artery diseases has not been fully clarified. Bai WW *et al.* found that TXL enhanced angiogenesis in the ischemic myocardium by up-regulating hypoxia-inducible factor 1α (HIF-1α) and VEGF expression, thereby improving ischemic myocardial function [14]. Considering the association between angiogenesis and plaque destabilization, we examined the effect of TXL on angiogenesis in advanced atherosclerotic plaques of apolipoprotein E deficient (apoE−/−) mice in a previous study. Different from the pro-angiogenesis in the ischemic myocardium, TXL attenuated VV neovascularization in advanced atherosclerotic lesions. Further we found that TXL inhibited inflammatory angiogenesis via the BMX/NF-κB/MAPK pathways in atherosclerotic lesions [15]. Thus, TXL might have different effects in different microenvironment. However, the effect of TXL on early atherogenesis is not clear.

In the present study, we treated cultured macrophages and apoE−/− mice with TXL to further explore the effect of TXL on VV proliferation and atherogenesis and the potential mechanisms.

## RESULTS

### TXL treatment reverses tumor necrosis factor-α induced expression of VEGF-A and ANGPT-1 in macrophages

**Figure 1:**
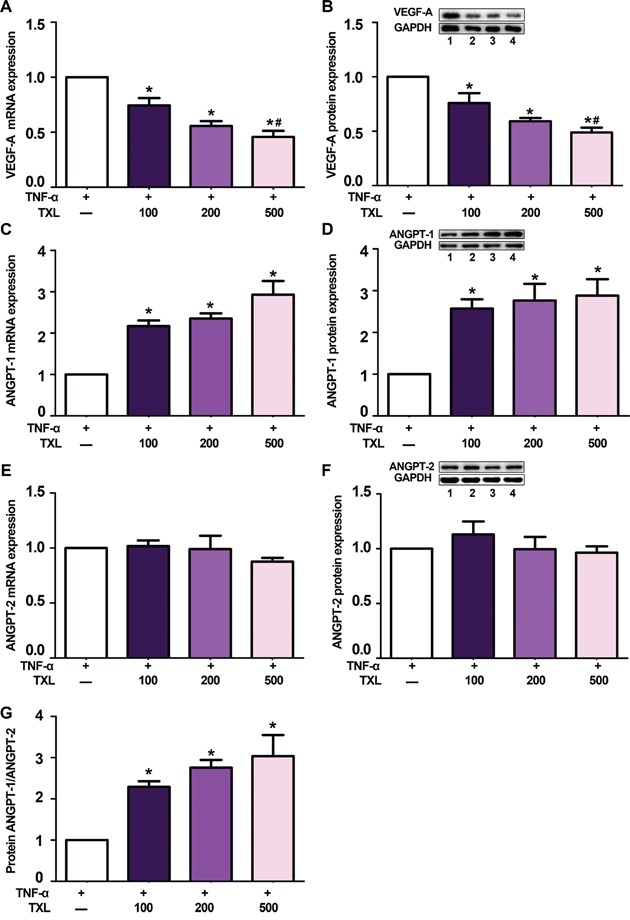
TXL solution regulates VEGF-A, ANGPT-1 and ANGPT-2 expression in TNF-α-stimulated RAW 264.7 cells **A.** qRT-PCR analysis of mRNA level of VEGF-A in TNF-α-stimulated RAW 264.7 cells pretreated with or without TXL solution (100, 200, 500 μg/ml). **B.** Representative western blot image and quantification of protein level of VEGF-A in TNF-α-stimulated RAW 264.7 cells pretreated with or without TXL solution. **C.** qRT-PCR analysis of relative mRNA level of ANGPT-1 in TNF-α-stimulated RAW 264.7 cells pretreated with or without TXL solution. **D.** Representative western blot image and quantification of protein level of ANGPT-1 in TNF-α-stimulated RAW 264.7 cells pretreated with or without TXL solution. **E.** qRT-PCR analysis of relative mRNA level of ANGPT-2 in TNF-α-stimulated RAW 264.7 cells pretreated with or without TXL solution. **F.** Representative western blot image and quantification of protein level of ANGPT-2 in TNF-α-stimulated RAW 264.7 cells pretreated with or without TXL solution. **G.** Quantification of the protein ratio of ANGPT-1/ANGPT-2 in TNF-α-stimulated RAW 264.7 cells pretreated with or without TXL solution. Bar 1: TNF-α only; bar 2: TNF-α+TXL 100 μg/ml; bar 3: TNF-α+TXL 200 μg/ml; bar 4: TNF-α+TXL 500 μg/ml; Data are mean ±SEM. * P<0.05 vs TNF-α only; # P<0.05 vs TNF-α+TXL 100 μg/ml.

In the present study, we first examined the effect of TXL on the expression of angiogenic factors *in vitro*. In RAW 264.7 cells, tumor necrosis factor-α (TNF-α) stimulated macrophages to secret VEGF-A; however, pre-treatment with TXL solution dose-dependently reduced both the mRNA and protein expression of VEGF-A (Figures 1A, 1B). In addition, compared to that of control, pre-treated with TXL solution markedly increased ANGPT-1 expression (Figures 1C, 1D), while ANGPT-2 expression showed no significant difference (Figures 1E, 1F). Thus, the protein ratio of ANGPT-1 to ANGPT-2 was increased after TXL pretreatment (Figure 1G).

### Intraplaque expression of VEGF-A is down-regulated while ANGPT-1 up-regulated in TXL treated mice

**Figure 2:**
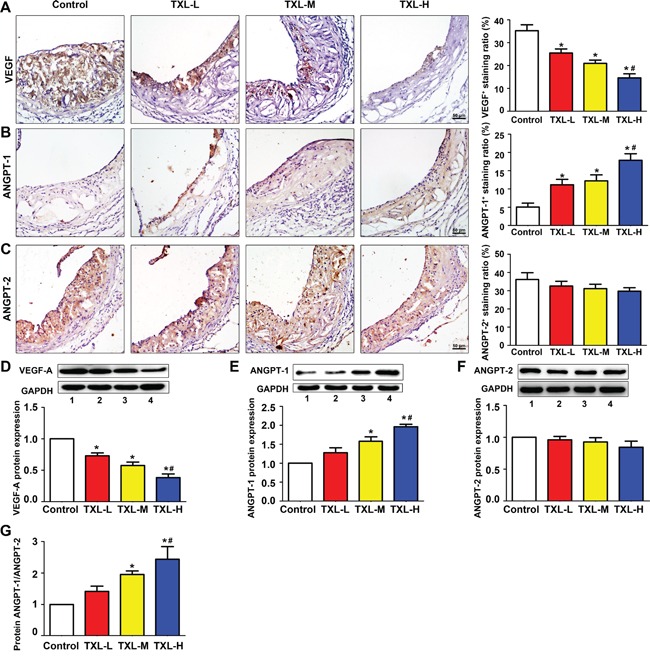
TXL regulates angiogenic factor expression in apoE−/− mice Representative immunohistochemical images and quantification of **A.** VEGF-A, **B.** ANGPT-1, **C.** ANGPT-2 levels in atherosclerotic plaques of apoE−/− mice. Representative western blot images and quantification of **D.** VEGF-A, **E.** ANGPT-1 and **F.** ANGPT-2 expression in treated mice aortas. **G.** Quantification of the protein ratio of ANGPT-1/ANGPT-2 in four groups of treated mice. Bar 1: control; bar 2: TXL-L; bar 3: TXL-M; bar 4: TXL-H; Data are mean ±SEM. * *P* <0.05 vs Control; ^#^ *P* <0.05 vs TXL-L.

On immunohistochemical analysis, the expression of VEGF-A was intense in the control group but was significantly down-regulated with TXL treatment, especially in high-dose TXL (TXL-H) group (Figure 2A). Although ANGPT-1 was weakly expressed in controls, its expression was increased with TXL treatment, especially in TXL-H group (Figure 2B). No significant difference in ANGPT-2 expression was found among the four treatment groups (Figure 2C). With TXL treatment, the protein level of VEGF-A was reduced and that of ANGPT-1 increased, with no change in ANGPT-2 level as compared with the control group (Figures 2D-2F). Accordingly, the protein ratio of ANGPT-1 to ANGPT2 in TXL-treated mice was in favor of ANGPT-1 (Figure 2G).

### TXL inhibits plaque-associated angiogenesis both *ex vivo* and *in vivo*

**Figure 3:**
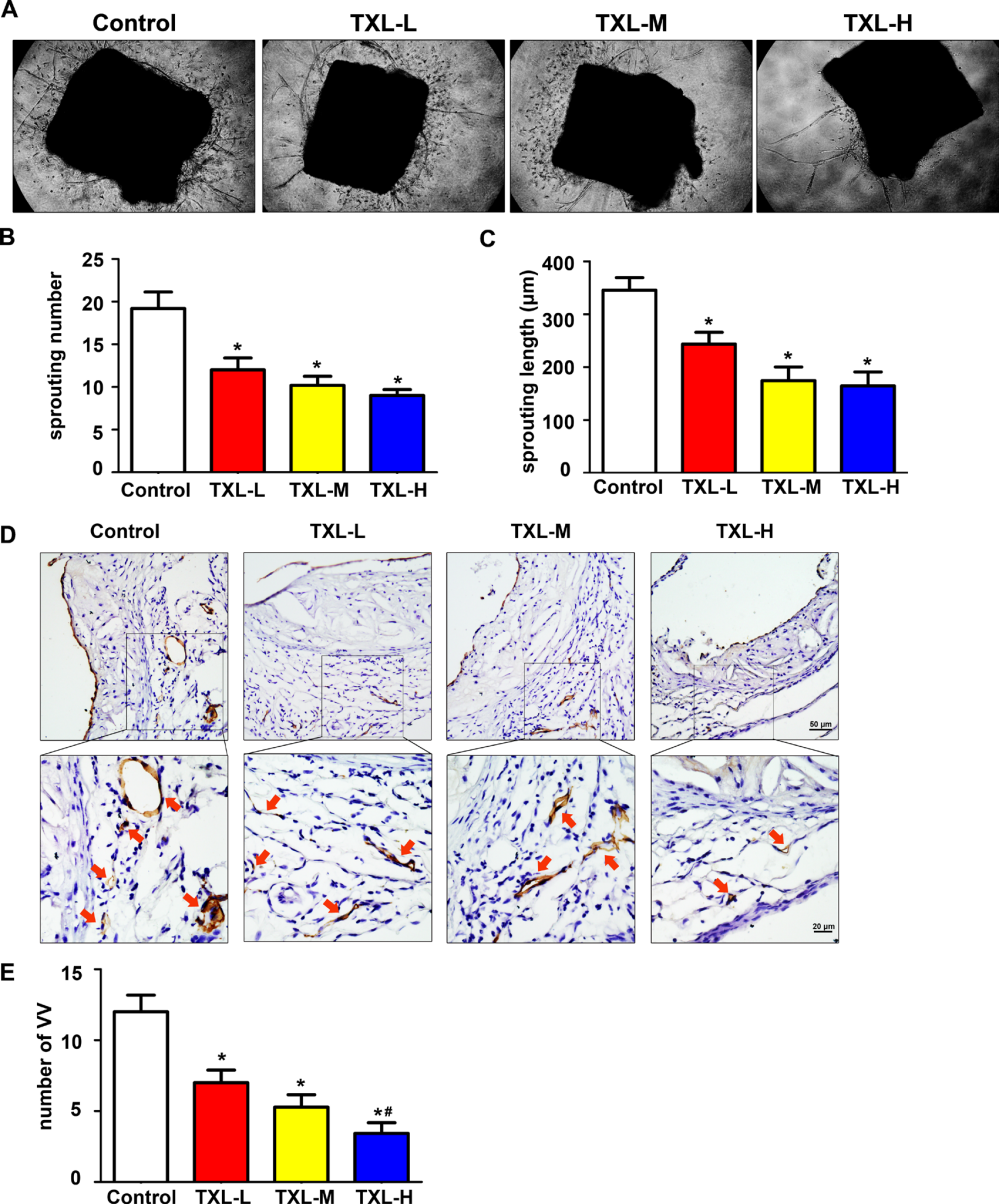
TXL inhibits plaque-associated angiogenesis ex vivo and *in vivo* **A.** Representative images of mouse aortic ring sprouts with four treatment groups. **B.** Quantification of sprouting number in aortic rings. **C.** Quantification of sprouting length in aortic rings. **D.** Representative images of VV proliferation by Tomato lectin staining in aortic root plaques. **E.** Quantitative analysis of VV number in four treatments. Data are mean ±SEM. * *P* <0.05 vs Control; ^#^ *P* <0.05 vs TXL-L.

Furthermore, we observed the effect of TXL on plaque-associated angiogenesis *ex vivo* by mouse aortic ring assay. The numbers and lengths of sprouting microvessel branches from aortic rings were reduced with TXL than control treatment (Figures 3A-3C). Then, we detected VV proliferation in atherogenesis *in vivo*. Commonly, VV were barely found in arteries of C57 BL/6J mice, but were enhanced in apoE−/− mice feeding high fat diet only for six weeks (Figure 3D, control). Interestingly, treatment with TXL significantly reduced VV numbers in adventitia surrounding the atherosclerotic plaques in aortic roots (Figures 3D, 3E).

### TXL suppresses early atherogenesis in apoE−/− mice

**Figure 4:**
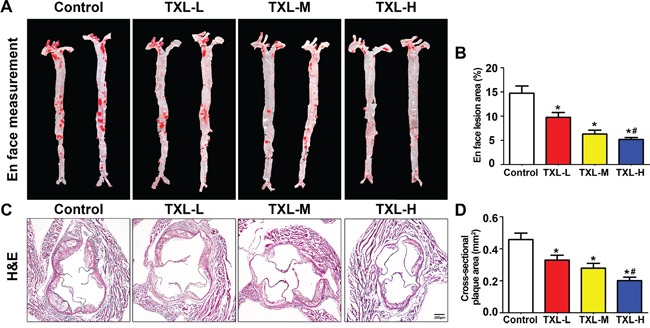
TXL suppresses early atherogenesis in apoE−/− mice **A.** Representative images of en face Oil-Red O staining of aortas in four groups of mice. **B.** Quantitative analysis of en face aorta lesions expressed as percentage lesion area relative to total aorta area. **C.** Representative images of cross-sectional aortic root lesions by H&E staining. **D.** Quantitative analysis of cross-sectional plaque area in aortic roots. Data are mean ± SEM. * *P* <0.05 vs Control; ^#^ *P* <0.05 vs TXL-L.

En face lesion analysis showed a significant decrease in plaque burden in the three TXL treatment groups, especially the TXL-H group, than in the control group (Figures 4A, 4B). Moreover, the cross-sectional plaque area of aortic roots was significantly reduced in the three TXL treatment groups than that of control, especially the TXL-H group (Figures 4C, 4D). However, no significant difference was found in serum lipid profiles including total cholesterol (TC), triglycerides (TG), low-density lipoprotein cholesterol (LDL-C) and high-density lipoprotein cholesterol (HDL-C) among the four groups of apoE−/− mice (Table 1). In addition, TXL treatment did not change body weight or serum glucose concentration as compared with the control group (Table 1).

**Table 1:** Body weight, serum lipid profiles and glucose concentration

Groups	BW (g)	TC (mmol/L)	TG (mmol/L)	HDL-C (mmol/L)	LDL-C (mmol/L)	glucose (mmol/L)
Control	27.77±0.30	30.14±1.67	2.16±0.14	7.78±0.18	3.81±0.39	8.873±0.30
TXL-treated						
Low dose	28.07±0.30	28.06±1.23	1.91±0.14	7.89±0.25	3.47±0.33	8.96±0.37
Medium dose	28.34±0.58	27.22±1.10	1.85±0.10	8.34±0.28	3.44±0.21	8.443±0.47
High dose	27.72±0.37	26.41±1.30	1.85±0.08	8.32±0.26	3.33±0.24	8.47±0.36
*p*	ns	ns	ns	ns	ns	ns

Data are expressed as mean±SEM; BW: body weight; TC: total cholesterol; TG: triglyceride; HDL-C: high-density lipoprotein cholesterol; LDL-C: low-density lipoprotein cholesterol; TXL: Tongxinluo; ns: not significant

### TXL stabilizes atherosclerotic plaque in apoE−/− mice

**Figure 5:**
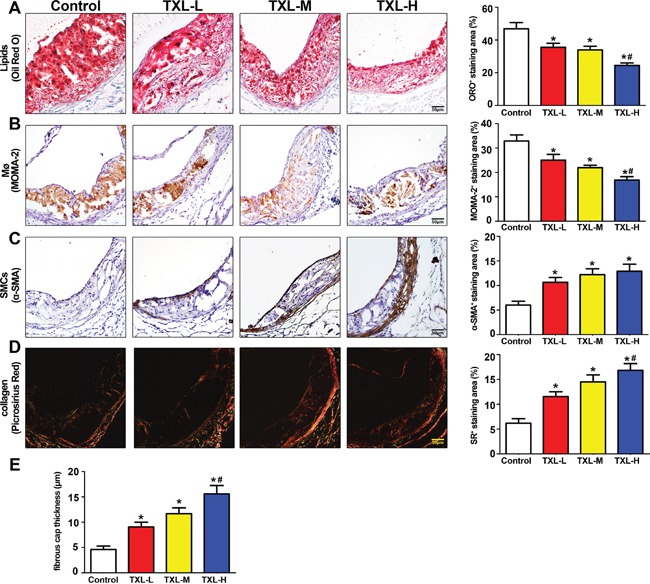
TXL stabilizes atherosclerotic plaque in apoE−/− mice **A.** Representative image and quantification of lipid deposition in four treatment groups by Oil-Red O staining. **B.** Representative immunohistochemical MOMA-2 staining image and quantification of plaque macrophage content. **C.** Representative immunohistochemical α-SMA staining and quantification of plaque smooth muscle cell content. **D.** Representative image and quantification of plaque collagen content by Picrosirius Red staining. **E.** Quantitative analysis of fibrous cap thickness by Picrosirius Red staining. Data are mean ±SEM.* *P* <0.05 vs Control; ^#^ *P* <0.05 vs TXL-L.

The intraplaque content of lipids and macrophages was significantly decreased in the three TXL treatment groups, especially the TXL-H group, than in the control group (Figures 5A, 5B). The content of SMCs in plaques was significantly increased in the three TXL treatment groups than in the control group (Figure 5C). Similarly, the plaque collagen content and fibrous cap thickness were significantly increased in the three TXL treatment groups, especially the TXL-H group, than in the control group (Figures 5D, 5E). Thus, TXL treatment dose-dependently changed the composition of plaques to a more stabilized phenotype.

## DISCUSSION

The principal finding of our study is that TXL treatment can decrease the expression of VEGF-A and increase that of ANGPT-1, while with no effect on ANGPT-2 expression, which resulted in a ratio of ANGPT-1 to ANGPT-2 in favor of ANGPT-1. Both *ex vivo* and *in vivo* study demonstrated that TXL treatment attenuated plaque-associated angiogenesis. On histological and morphological analysis, TXL treatment dose-dependently reduced the plaque burden, plaque size and changed the composition of plaques to a more stabilized phenotype. Thus, TXL treatment might mitigate atherogenesis through regulating angiogenic factors expression and inhibiting VV proliferation in atherosclerotic plaques, which shed new light on the anti-atherosclerotic effect of TXL.

TXL is a traditional Chinese medication that has been widely used in clinic for cardiovascular diseases for decades [13, 16–18]. With increasing investigations of TXL, more effects were found, including lipid-lowering, anti-inflammation and enhancing cardiac micro-vascular endothelial barrier function [19, 20]. In a murine myocardial infarction model, TXL enhanced angiogenesis in the ischemic myocardium by up-regulating HIF-1α and VEGF expression, thereby improving ischemic myocardial function [14]. Increasing evidence suggests an association of VV expansion and intra-plaque neovascularization with atherosclerosis progression and instability [21–23]. Thus, we examined the effect of TXL on angiogenesis in advanced plaques of apoE−/− mice. In contrast to the pro-angiogenic effect in the ischemic myocardium, TXL treatment substantially attenuated VV neovascularization in murine advanced atherosclerotic lesions by inhibiting inflammatory angiogenesis via BMX/NF-κB/MAPK pathways, leading to enhanced plaque stabilization [15]. These data indicated that TXL may exert an opposite effect on angiogenesis in different microenvironments. However, little is known about the role of TXL on early atherogenesis in apoE−/− mice.

VV neovascularization contributes to atherogenesis, and its extent and distribution is rigorously regulated by angiogenesis-related factors. The VEGF family members represent potent mitogenic and pro-migratory factors for endothelial cells [24–28]. The expression of VEGF-A, the major VEGF subtype [29, 30], was higher in unstable than stable carotid plaques derived from human carotid thromboendarterectomy samples [11]. Moreover, VEGF promoted atherosclerosis progression in both apoE/apoB^100^ double-deficient mice and rabbits [7]. The angiopoietin/Tie system is another important family in regulating angiogenesis in atherogenesis. ANGPT-1 can block VEGF-induced vascular permeability and stabilize the interaction between endothelial cells and surrounding support cells, whereas ANGPT-2, considered as an antagonist of ANGPT-1, leads to vascular destabilization or regression [8–10]. Indeed, in human carotid thromboendarterectomy samples, reduced ANGPT-1 and increased ANGPT-2 levels were observed in unstable plaques [11]. The ratio of ANGPT-1 to ANGPT-2 in favor of ANGPT-2 was detected in the atherosclerotic plaques with high microvessel density as well as hemorrhagic plaques [12, 31]. ANGPT-2 blocking antibodies were shown to reduce early atherosclerosis development in hypercholesterolemic mice but had no effect on the size or composition of pre-existing plaques [32]. Despite controversial results demonstrating overexpression of ANGPT-2 decreased plaque formation in apoE−/− mice by inhibiting oxidation of LDL [33], the transgenic overexpression of ANGPT-2 could not mimic the role of endogenous ANGPT-2. In the present study, TXL treatment dose-dependently reduced TNF-α-induced VEGF expression and increased ANGPT 1 expression *in vitro*. In addition, control mice showed higher expression of VEGF and lower expression of ANGPT-1 than TXL-treated mice, whereas no significant difference in the expression of ANGPT-2 was detected. As a result, the ratio of ANGPT-1 to ANGPT2 in TXL-treated mice was in favor of ANGPT-1. These results agreed with immunohistological staining findings in human carotid endarterectomy samples. Thus, angiogenesis regulation is far more complicated than previously recognized and inhibiting or promoting one or two angiogenic factors is not sufficient [34, 35]. Moreover, increasing preclinical and clinical studies demonstrated that anti-angiogenic drugs, such as bevacizumab for treating cancers or retinal vascular diseases, can cause adverse effects in healthy vasculature [36–38]. TXL treatment may serve as an alternative anti-angiogenic therapy due to its pleiotropic effects and a high safety profile.

The newly formed VV, characterized as deteriorated inter-endothelial junctions and lacking of pericytes coverage, are considered as conduits for cellular and soluble lesion components to favor progression and complications of atherosclerosis such as intra-plaque hemorrhage and further rupture [39]. However, increasing studies have demonstrated that neovascularization is closely associated with early atherogenesis [3–5]. In the porcine experimental hypercholesterolemia model, treatment with the anti-angiogenic drug thalidomide inhibited VV proliferation and neointimal formation in early atherosclerosis [5]. Likewise, in the present study with apoE−/− mice, inhibition of VV proliferation with TXL treatment contributed to reduced atherosclerotic plaque formation and enhanced plaque stabilization.

Taken together, TXL treatment could mitigate plaque formation at the initial stage of atherosclerosis and stabilize atherosclerotic plaques in apoE−/− mice. Inhibiting VV proliferation and regulating the expression of angiogenic factors might be a therein crucial part.

## MATERIALS AND METHODS

### Ethics statement

All animal experimental protocols and animal care procedures complied with the ethical standards and according to the Animal Management Rule of the Ministry of Public Health, People's Republic of China (documentation No 55, 2001) and were approved by the Animal Care Committee of Shandong University.

### TXL ultrafine powder and solution preparation

We obtained TXL ultrafine powder from Shijiazhuang Yiling Pharmaceutical Co. (Hebei, China). The herbal components of TXL were authenticated and standardized according to marker compounds complying with the Chinese Pharmacopoeia 2005. TXL ultrafine powder and solution were prepared as described [15].

### Cell culture

RAW 264.7 cells were purchased from the American Type Culture Collection (ATCC, VA, USA). Cells were cultured in Dulbecco's modified Eagle medium (DMEM) containing 10% fetal bovine serum (FBS; Invitrogen, CA, USA). The reagent of TNF-α was from PeproTech (NJ, USA). Before stimulation with TNF-α, RAW 264.7 cells were pre-incubated with or without TXL solution (100, 200, 500 μg/ml) for 24 hr.

### Quantitative real time PCR (qRT-PCR)

After stimulation with TNF-α (10 ng/ml), RNA was extracted from treated RAW 264.7 cells by using Trizol reagent (Invitrogen). cDNA synthesis involved use of the PrimeScript RT reagent Kit with gDNA Eraser (TakaRa Biotechnology, Dalian, China). The mRNA expression of VEGF-A, ANGPT-1 and ANGPT-2 was quantified by qRT-PCR with SYBR Green qPCR Master Mix reagent (Takara Biotechnology). Glyceraldehyde 3-phosphate dehydrogenase (GAPDH) was used as an internal control. The sequences of forward and reverse primers for VEGF-A, ANGPT-1, ANGPT-2 and GAPDH are in Table 2. The 2^−ΔΔCT^ method was used to analyze relative expression between treatments [40]. The results were normalized against GAPDH expression.

**Table 2:** Primer sequences for qRT-PCR analysis

Primers	sequences 5′-3′
**VEGF-A**	
Forward	CTTGTTCAGAGCGGAGAAAGC
Reverse	ACATCTGCAAGTACGTTCGTT
**ANGPT-1**	
Forward	CACATAGGGTGCAGCAACCA
Reverse	CGTCGTGTTCTGGAAGAATGA
**ANGPT-2**	
Forward	CCTCGACTACGACGACTCAGT
Reverse	TCTGCACCACATTCTGTTGGA
**GAPDH**	
Forward	AGGTCGGTGTGAACGGATTTG
Reverse	TGTAGACCATGTAGTTGAGGTCA

VEGF-A: vascular endothelial growth factor A; ANGPT-1: angiopoietin 1; ANGPT-2: angiopoietin 2; GAPDH: glyceraldehyde 3-phosphate dehydrogenase

### Animal preparation and protocols

A total of 100 apoE−/− mice (male, 12 weeks old) were obtained from Peking University (Beijing, China). Mice were housed in cages with food and water given ad libitum in a temperature-controlled facility with a 12-hr light/12-hr dark cycle. All mice were fed a high-fat diet (0.25% cholesterol and 15% cocoa butter) for the entire protocol. One week later, mice were randomly divided into four groups (n=25 per group) for treatment: control, an equal volume of saline; and low-dose TXL (TXL-L), medium-dose TXL (TXL-M) and high-dose TXL (TXL-H), with TXL given orally at 0.38, 0.75, 1.5 g/kg/d, respectively, for five weeks. At the end of six weeks, all animals were weighed, and then underwent euthanasia.

### *Ex vivo* aortic ring angiogenesis assay

After five weeks treatment, aortas of mice in each group were dissected for aortic ring assay as described with some modifications [41]. Briefly, dissected thoracic aortas from treated apoE−/− mice were cut into about 0.5-mm long rings, washed with serum-free medium and embedded in rat type I collagen (Millipore, MA, USA). Aortic rings were incubated with endothelial cell medium and the medium was changed on days 3, 6 and 8. Pictures were captured by use of a Canon Camera linked to a light microscope on day 8 and the number of microvessels sprouting was counted and their length measured.

### Biochemical measurement

At the end of the experiment, mice fasted overnight and blood samples were collected from the apex of the left ventricle. Serum lipid profiles, including TC, TG, LDL-C and HDL-C, and glucose concentration were measured by enzymatic assay with use of an automatic biochemical analyzer (Roche Cobas Integra 800, Basel, Switzerland).

### Tissue preparation and collection

After mice were anesthetized by phenobarbital and blood was collected, mice were rapidly perfused with 0.9% saline, then underwent perfusion fixation with ice-cold 4% paraformaldehyde or were frozen by using of liquid nitrogen for molecular biological study. The heart, aorta, liver and kidney were collected. Tissues were fixed in 4% paraformaldehyde overnight. Aortic roots were embedded in optimum cutting temperature (OCT) compound for cryosections. Serial cross-sections 5-μm thick of the aortic root were collected and stored at −20°C.

### VV staining

To detect the VV in adventitia, endothelial cells were stained by perfusion of biotinylated Lycopersicon esculentum (Tomato) lectin (Vector, CA, USA) as described [23]. Briefly, each mouse was perfused with 0.9% saline, 1% paraformaldehyde and 0.5% glutaraldehyde, 1% FBS in saline, 200 μg biotinylated tomato lectin in 1% FBS, 1% FBS in saline, and at last 0.9% saline for 3 min each in sequence. Then the aorta was collected with some perivascular tissues and fixed in methanol at 4°C overnight. Aortic roots were embedded in OCT on the second day and cryosections 5-μm thick were collected and stored at −20°C. Tomato lectin distribution representing endothelial cells was detected by avidin-biotin complex and horseradish peroxidase (HRP) substrate (Vector).

### Histology and immunohistochemistry analysis

To assess overall burden and distribution of atherosclerosis, en face lesion staining with Oil-Red O was performed as previously described [15, 42]. Cross-sections of the aortic roots (predilection site of atherosclerosis) were stained with hematoxylin and eosin (H&E) following a standard protocol of our lab. The content of lipids and collagen of aortic plaques was detected by Oil-Red O staining and Picrosirius red staining, respectively. The immunohistochemical staining procedure was as previously described [15, 42]. Targeted proteins were identified by the following antibodies against macrophages/monocytes antigen (MOMA-2, 1:150, AbD Serotec, Oxford, UK), α smooth muscle actin (α-SMA, 1:200, Abcam, Cambridge, UK), ANGPT-1 (1:100, Abcam), ANGPT-2 (1:200, Abcam), and VEGF-A (1:150, Millipore).

Histological and immunohistochemical staining was analyzed by using Image-Pro Plus 6.0 (IPP 6.0, Media Cybernetics, MD, USA). The en face analysis of aortas was performed as previously described [15, 42]. Plaque sizes were analyzed by H&E staining of the cross-sectional aortic sinus. Lipid deposition and content of collagen, macrophages, SMCs and angiogenic factors were analyzed as the ratio of the positive staining area of targeted protein to total plaque area. Fibrous cap thickness was evaluated by Picrosirius red staining as described [38, 42].

### Western blot analysis

Proteins were isolated from treated RAW 264.7 cells and mice aortas. Tissues were lysed in lysis buffer (100 mM Tris-Cl, pH 6.8, 4%(m/v) SDS, 20% (v/v) glycerol, 200 mM β-mercaptoethanol, 1 mM PMSF, and 1 g/ml aprotinin) and proteins were transferred to PVDF membranes, which were then incubated with primary antibodies for VEGF-A (1:500, Millipore), ANGPT-1 (1:500, Abcam), ANGPT-2 (1:1000, Abcam), and GAPDH (1:5000, Sigma-Aldrich, Louis, USA) overnight at 4°C. The membrane bands were visualized by use of chemiluminescence (Millipore) and quantified by densitometry.

### Statistical analysis

Data are expressed as mean ± SEM and analyses involved use of GraphPad Prism 5 (La Jolla, CA, USA). Differences among groups were analyzed by one-way ANOVA followed by Tukey post-hoc test. Statistical significance was defined as *P* <0.05.
